# MiR-1 mediates autophagy via ATG14 in sheep Leydig cells infected with *Brucella melitensis* strain BA0711

**DOI:** 10.3389/fvets.2026.1809720

**Published:** 2026-06-16

**Authors:** Zitong Zhang, Junming Jiang, Yiwen Cheng, Shihua Niu, Hejie Qian, Yujing Fu, Yimei Chen, Mengqi Zhang, Qiaoling Chen, Hongyan Gao, Churiga Man, Li Du, Si Chen, Fengyang Wang

**Affiliations:** Hainan Key Laboratory of Tropical Animal Reproduction & Breeding and Epidemic Disease Research, School of Animal Science and Veterinary Medicine, Hainan University, Haikou, China

**Keywords:** autophagy, brucellosis, miRNAs, mRNA, sheep Leydig cells

## Abstract

*Brucella* spp. achieve intracellular parasitism by hijacking host autophagy pathways. Sheep Leydig cells (SLCs) are important intracellular parasitic target cells for *Brucella melitensis* (*B. melitensis*). To investigate the molecular regulatory network induced by *B. melitensis* in SLCs, particularly whether the autophagic pathway response differs from that in other cell types, transcriptome sequencing analysis was performed on SLCs treated with *B. melitensis* BA0711. According to the criteria of |log_2_FC| > 0.582 and *p* < 0.05, a total of 6,036 differentially expressed genes (DEGs) and 100 differentially expressed miRNAs (DE miRNAs) were identified, both of which were significantly enriched in the autophagy–animal pathway. RT-qPCR confirmed that the expression trends of validated DEGs were consistent with mRNA sequencing results. In the autophagy–animal pathway, *ULK1*, *ATG13*, *ATG14*, *PIK3C3*, *ATG5*, *ATG101*, and *ATG16L2* were predicted as the top seven hub DEGs. Overexpression and knockdown experiments of miR-1, together with dual-luciferase reporter assays, demonstrated that miR-1 inhibits the mRNA expression of its downstream target ATG14 by binding to the 3’UTR of ATG14. This study revealed the potential regulatory role of the miR-1–ATG14 axis in this process. These findings provide a novel, cell-specific perspective on the early host–pathogen interactions elicited by *B. melitensis* within a tissue microenvironment relevant to brucellosis, offering a foundation for future mechanistic studies in more complex systems.

## 1 Introduction

*Brucella* spp. are facultatively intracellular and Gram-negative coccobacilli, comprising at least 11 species (1). These pathogens invade hosts (including both livestock and humans) mainly through mucosal surfaces, inducing localized or systemic infections termed brucellosis (2, 3). The virulence factors of *Brucella* promote immune evasion via diverse strategies, such as interference with pattern recognition receptors, regulation of autophagy and apoptosis, and suppression of antigen presentation (4). Notably, *Brucella* utilizes autophagy-initiation proteins to form *Brucella*-containing autophagic vacuoles, facilitating the completion of its intracellular life cycle and intercellular transmission (5). Brucellosis in livestock primarily damages the reproductive system, leading to conditions such as orchitis, epididymitis, placentitis, endometritis, late abortion, weak offspring, and stillbirth (6). Based on integrated epidemiological-economic prediction models, the estimated annual economic losses per infected pastoral cattle, sheep, and goat in northern and central Tanzania were 26.2–211.7 (median 74.4), 3.4–23.1 (median 9.7), and 3.7–25.0 (median 10.6) US dollars, respectively (7). Studies from Kazakhstan employing macro-level cost accounting have indicated that annual expenditures for brucellosis control, particularly for animal testing and compensation, reach 45 million US dollars, highlighting the substantial economic burden imposed by the disease (8). Vaccination represents the most cost-effective strategy for disease control. The development of vaccines, from classical strains such as *Brucella abortus* S19 (*B. abortus* S19), *Brucella abortus* RB51 (*B. abortus* RB51), and *Brucella melitensis* Rev.1 (*B.melitensis* Rev.1) to novel candidates including *Brucella melitensis* BA0711 (*B.melitensis* BA0711) and mRNA vaccines, reflects the continuous deepening of our understanding of its pathogenic and immune mechanisms (9, 10).

Within this context, microRNAs (miRNAs) serve as key post-transcriptional regulators, playing a central role in the interaction between *Brucella* and its host. It is particularly important that pathogenic wild-type strains and attenuated vaccine strains, due to differences in their virulence factors and intracellular survival strategies, guide host cells to form distinctly different miRNA expression profiles. For instance, in macrophages infected with the wild-type *B. suis* 1,330 strain, miR-155 and miR-21-5p participate in the regulation of the inflammatory response pathway by suppressing the production of IL-12 and TNF-*α*, thereby promoting bacterial survival (11, 12). Meanwhile, miR-24 participates in the regulation of the host cell DNA surveillance pathway by downregulating *STING* expression, thereby impairing the recognition of *Brucella* DNA (13). In contrast, miR-3068-5p and other miRNAs participate in the regulation of innate immune activation pathways by inhibiting negative regulators of the NF-κB pathway, thereby assisting the host in resisting infection (14, 15).

Further research indicates that *B. melitensis* can induce highly specific miRNA regulatory networks in different types of host cells. In macrophages infected with the vaccine candidate strain M5-90-*ΔOmp25*, miR-146a-5p and miR-155-5p participate in the regulation of immune response pathways through their differential expression (16). In contrast, infection with the M5-90-*Δper* strain leads to significant upregulation of miR-146b-5p, which participates in the regulation of autophagy by targeting and inhibiting *Tbc1d14* expression (17). These miRNA expression patterns induced by vaccine strains differ markedly from those triggered by wild-type strains (e.g., 16 M) (13). More interestingly, in goat fibroblasts infected with the *B. melitensis* M5-90 vaccine strain, miR-744 and miR-29a-5p participate in the regulation of NF-κB and interferon signaling pathways by targeting *NFKB1* and *IFNAR2* genes, respectively, demonstrating a completely distinct expression profile from that observed in macrophages (18). This strongly suggests that the immunoprotective mechanisms of vaccine strains and their impact on host cell functions may depend on the unique miRNA-mRNA regulatory networks established between the strain and specific cell types.

As a critical intracellular niche for *B. melitensis*, Leydig cells play essential roles in spermatogenesis and androgen production while serving as key components of the testicular immune microenvironment (19–21). Recent work by Chen et al. employed single-cell transcriptomics to systematically map the dynamic reprogramming of testicular immunity in *Brucella*-infected goat testes, revealing hallmark events including T-cell hyperactivation and alterations in pivotal signaling pathways (22). Evidence further indicates that live vaccines can modulate autophagy in macrophages via specific molecules such as miR-146b-5p (23). Nevertheless, how live *Brucella* vaccines (such as BA0711) influence cellular functions–particularly autophagy–through differential miRNA regulatory networks in these reproductive cell populations remains poorly explored. Given the established role of autophagy in the intracellular lifecycle of *Brucella* (5), it is still unclear whether the live vaccine strain BA0711 employs a distinct, potentially autophagy-related miRNA network in SLCs. To address this, we used SLCs as an *in vitro* model infected with the BA0711 vaccine strain. By integrating high-throughput sequencing and molecular biology techniques, we systematically profiled the miRNA expression landscape in these reproductive target cells post-infection. Further validation through molecular interaction assays and pathway enrichment analysis allowed us to delineate the autophagy-related networks regulated by these miRNAs, thereby advancing the molecular understanding of microbe-host cell interactions.

## 2 Materials and methods

### 2.1 Cells and strain

Immortalized SLCs (commercially designated as immortalized sheep testes interstitial cells) were purchased from Hefei Wanwu Bioterch, New Zealand) at 37 °C and 5% CO_2_ using an electric thermostatic incubator (DNP-9082, Shanghai Jinghong Experimental Equipment Co., Ltd., Chichnology Co., Ltd. (Hefei, China) and cultured using Dulbecco’s Modified Eagle Medium (DMEM, Biosharp, Hefei, China) supplemented with 10% (*v*/*v*) fetal bovine serum (FBS, NEWZERUM Ltd., Christchuna). BA0711 was obtained from Chongqing Aolong Biological Products Co., Ltd. (Hohhot, China) and all related experiments were conducted in a biosafety laboratory. It was identified through PCR identification (24) using bacterial cultures grown in BBL™ *Brucella* Broth (Becton, Dickinson and Company, Franklin Lakes, NJ, USA) at 37 °C for 72 h.

### 2.2 Bacterial stimulation

SLCs were seeded in 10 cm cell-culture dishes (*n* = 6, 3 × 10^6^ cells per dish), which were evenly divided into two groups. The cells were cultured as described above for 4 h. Sterile phosphate-buffered saline (Biosharp, Hefei, China) resuspended BA0711 (MOI = 100 (18) grown in logarithmic growth phase) was added to the cells of the experimental group (EBA12). Meanwhile, an equal amount of PBS was added to the cells of the control group (CK). After thorough mixing, the cells were placed in incubator for 4 h. The supernatant of cell cultures were discarded and cells were rinsed thrice using PBS. Subsequently, 8 mL DMEM supplemented with 50 μg/mL gentamicin (Beyotime Biotech Co., Ltd., Shanghai, China) was added in each dish to remove extracellular bacteria. One hour later, the medium was replaced with fresh DMEM containing 10% FBS (without gentamicin). After continuous culture for 12 h (25), the supernatant was collected for bacterial identification. Trizol (Tiangen Biotech Co., Ltd., Beijing, China) was added to the PBS-rinsed cells and mixed thoroughly for total RNA extraction according to the manufacturer’s instructions.

### 2.3 RNA sequencing

The mRNA library was constructed using the Hieff NGS® Ultima Dual-Mode mRNA Library Prep Kit (Yeasen Biotechnology Co., Ltd., Shanghai, China) following the manufacturer’s protocol. Briefly, mRNA was enriched from qualified total RNA via poly(A) selection using kit-provided beads, followed by fragmentation via heat treatment to generate 200–300 nt fragments. The fragmented mRNA was reverse transcribed to synthesize the first cDNA strand, and the second strand was synthesized with simultaneous end repair and 3′-dA tailing. Illumina X plus platform (Illumina, Inc., San Diego, CA, USA) adapters were ligated, and the products were size-selected using Hieff NGS^®^ DNA Selection Beads. The library was then amplified by PCR and purified, completing library construction. For the miRNA library, a separate aliquot of qualified total RNA was processed using the NEBNext^®^ Multiplex Small RNA Library Prep Set for Illumina^®^ (New England Biolabs, Inc., Ipswich, MA, USA) according to the manufacturer’s instructions, as previously described. The purified small RNA (predominantly miRNA) was obtained by excising the 18–30 nt fraction from 15% denaturing polyacrylamide gel electrophoresis (PAGE) of total RNA. Library construction proceeded as follows: small RNA was ligated with Illumina-compatible 3′ and 5′ adapters, reverse transcribed, amplified by PCR, and subjected to precise size selection via PAGE (6% or 8% native gel) to recover 140 bp fragments. The final library was eluted in elution buffer. Eventually, the qualified and quantified miRNA library was sequenced on the same platform.

### 2.4 mRNA sequencing analysis

The clean reads of mRNA sequencing were derived from filtering the raw reads by fastp (v0.18.0) (26). Among them, the reads matched to rRNA were removed by Bowtie2 (v2.2.8) (27), and then aligned with the *Ovis aries* reference genome (GCF_016772045.2) using HISAT2 (v2.1.0) (28). StringTie (v1.3.4) was applied for transcript assembling based on the successfully aligned sequences (29). Afterwards, RSEM (v1.2.19) was utilized for the normalization of the expression levels of all genes in each sample (30). Based on the normalized expression data (transcripts per million, TPM), we performed Principal Component Analysis (PCA) and generated a correlation heatmap using R^1^ to visualize differential gene expression between groups. Differential gene expression between groups was analyzed using DESeq2 (v1.20.0) on the raw count data (31). Differentially expressed genes (DEGs) were identified with |log_2_FC| > 0.582 and *p* < 0.05 as the standard. These DEGs were subsequently annotated in Gene Ontology (GO) database (GO.db v3.14.0) and Kyoto Encyclopedia of Genes and Genomes (KEGG) enrichment analysis was performed using in-house scripts, with the hypergeometric test implemented *via* the statspackage (v3.2.1) in R. The GO and KEGG analyses were performed on the set of differentially expressed genes identified using the thresholds of |log_2_FC| > 0.582 and *p* < 0.05. Furthermore, we predicted the protein–protein interaction (PPI) network for the 61 differentially expressed genes (DEGs) in the selected pathway (the gene list is provided in Supplementary Table S1). This prediction was performed using the STRING database (accessed on September 23, 2025) with the highest confidence threshold (0.900), and the resulting interaction relationships are summarized in Supplementary Table S2. Finally, the constructed PPI network was visualized using Cytoscape software (version 3.10.3) (32, 33). The key subnetworks and hub proteins were screened by the edge percolated component (EPC) algorithm within the cytoHubba plugin.

### 2.5 miRNA sequencing analysis

All of the clean tags were aligned with small RNAs in GeneBank database and Rfam database to identify and remove rRNA, scRNA, snoRNA, snRNA, and tRNA. Known and novel miRNAs of *Ovis aries* were identified by Bowtie (v1.1.2) and MiRdeep2 (v0.0.7), respectively (33). According to the standards of |log_2_FC| > 0.582 and *p* < 0.05, DE miRNAs between CK and EBA12 were screened using edgeR (v3.12.1) (34). In addition, Miranda (v3.3a) and TargetScan (v7.0) were used to predict the target genes of DE miRNAs. GO and KEGG enrichment analyses were performed on these genes following the methods applied in mRNA sequencing analysis. MiRNA-gene regulatory relationship was constructed based on DE miRNA-DEG pairs in the selected pathway using Cytospace.

### 2.6 RT-qPCR validation

To verify sequencing results, we performed RT-qPCR to detect the expression of the selected DEGs and DE miRNAs. Primers were designed and listed in Supplementary Tables S3, S4. For DEGs validation, each qualified RNA (1 μg) was reverse transcribed into cDNA by FastKing gDNA Dispelling RT SuperMix (Tiangen Biotech Co., Ltd., Beijing, China). Subsequently, the cDNA (1:3 dilution) served as the template for RT-qPCR assays of DEGs, which were performed using SYBR^®^ Green Premix Pro Taq HS qPCR Tracking Kit (Rox Plus, Accurate Biotechnology Co., Ltd., Changsha, China) on QuantStudio™ 5 Real-Time PCR System (Applied Biosystems Inc., Waltham, MA, USA). For DE miRNAs validation, total RNA was reverse transcribed into cDNA with specific primers (Supplementary Table S5). Afterwards, the RT-qPCR assays were conducted using SYBR^®^ Green qPCR Master Mix (ROX plus, Arraystar Inc., Rockville, MD, USA) on QuantStudio™ 5 Real-Time PCR System. Relative expression levels of DEGs and DE miRNAs were calculated using the 2^(−ΔΔCt)^ method, with *GAPDH* and snRNA U6 used as internal reference genes for mRNA and miRNA quantification, respectively. The results were represented as mean ± standard error and plotted using GraphPad Prism8. Unpaired T-tests were performed to evaluate the statistically significant difference. *, **, ***and **** indicate the value of *P* was less than 0.05, 0.01, 0.001, 0.0001, respectively, and ns represents no significant difference.

### 2.7 miRNA-gene regulation validation

To determine whether the DE miRNAs regulate the DEGs in the selected pathway, miRNA mimics or inhibitors (Supplementary Table S6) were transfected into SLCs, respectively, using Lipofectamine™ RNAiMAX Reagent (Thermo Fisher Scientific) according to the manufacturer’s instructions. Briefly, SLCs were seeded in 12-well plates (1.3 × 10^5^ cells/well) 12 h prior to transfection. For each well, 100 pmol of RNA oligos (miRNA mimics or inhibitors) and 3 μL Lipofectamine™ RNAiMAX were separately diluted in 100 μL Opti-MEM (Biosharp, Hefei, China), then they were mixed and incubated for 20 min at room temperature to form transfection complex. Subsequently, the transfection complex, along with 800 μL Opti-MEM, was added to cells at 50%–60% confluence. The medium was replaced with 1 mL DMEM supplemented with 10% FBS 6 h post-transfection. Transfection efficiency was confirmed by fluorescence microscopy using parallel transfections with Cy3-labeled or FAM-labeled scrambled RNA oligos. Untreated SLCs served as the negative control (NC). At 48 h post-transfection, total RNA was extracted using the FastPure^®^ Cell/Tissue Total RNA Isolation Kit (Vazyme Biotech Co., Ltd., Nanjing, Jiangsu, China) and reverse-transcribed into cDNA. RT-qPCR assays for the target genes of DE miRNAs were performed as described above.

### 2.8 Dual-luciferase reporter assay

Target gene prediction was performed using the MiRanda (v3.3a) and TargetScan (v7.0) databases, revealing the potential binding site between miR-1 and the 3′-UTR of *ATG14*. Primers were designed to amplify the predicted binding region in the 3′-UTR of *ATG14*: forward primer ATG14f (5′-GAGGACAGGCATGGACCCCA-3′) and reverse primer ATG14r (5′-GCACATGGAGTGGGTGGTAGG-3′). High-fidelity PCR amplification was conducted using cDNA reversely transcribed from RNA of SLCs as the template. After digestion by two restriction endonucleases (*SacI* and *NheI*), the amplified fragment was ligated into the pGL3-Basic vector to construct the luciferase reporter plasmid pGL3-Basic-ATG14 WT. In addition, the mutant plasmid lacking the miR-1 binding site, pGL3-Basic-ATG14 MUT, was constructed by *de novo* synthesis. The SLCs were seeded in 24-well plate (2 × 10^4^ cells per well) and three wells of biological replicates were set for each group. After transfection with miR-1 mimics or NC mimics for 48 h, the fluorescence intensity was detected by Dual-Luciferase Reporter Assay System (E1910, Promega) using Microplate reader (JW-QA34, Tecan). The data were calculated by the formula: Ralative Luciferase Activity = Firefly/Renilla. Transfection efficiency was calibrated based on pRL-TK internal reference plasmid. Data analysis was performed using GraphPad Prism 9.0 for t-test. The results were represented as mean ± standard error.

## 3 Results

### 3.1 SLCs treated with *Brucella melitensis* BA0711

**Figure 1:**
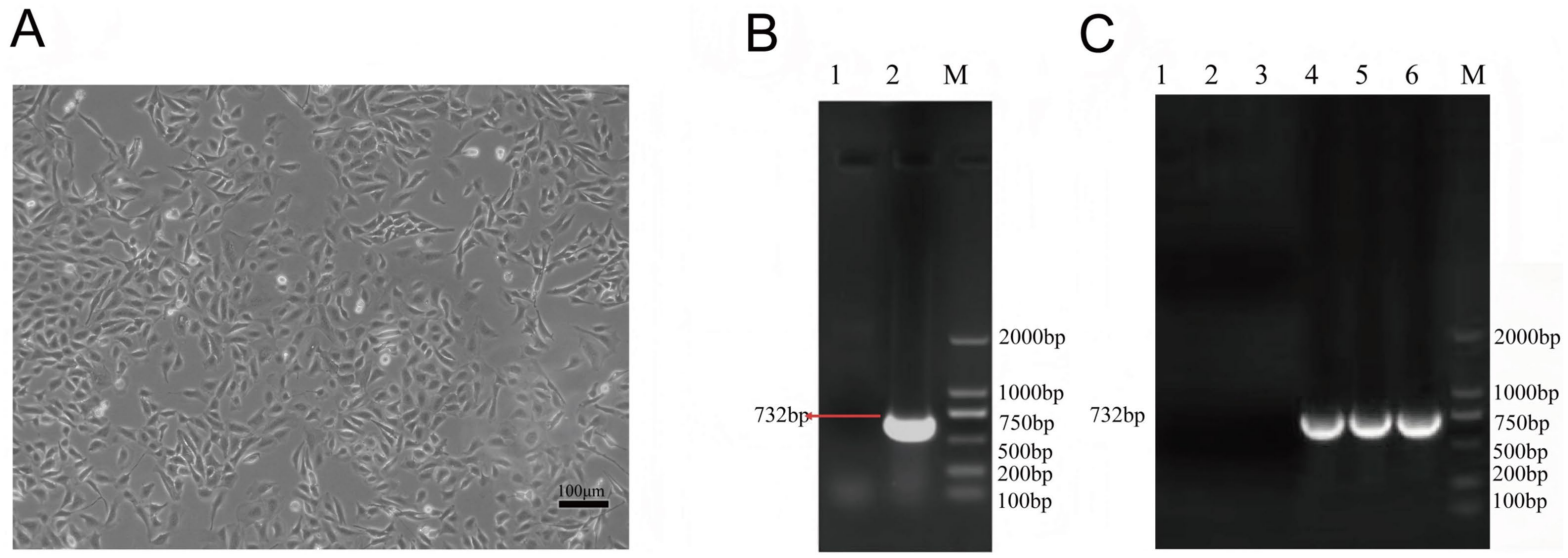
Exposure of SLCs to BA0711 and PCR verification. **(A)** Morphology of *in vitro*-cultured SLCs (Scale bar = 100 μm). **(B)** Species verification of BA0711 by *B. melitensis*-specific PCR. Lane 1, negative control. Sterile ddH_2_O was added in the PCR system instead of bacterial solution. Lane 2, BA0711. Lane M, D2000 maker. **(C)** PCR identification of *B. melitensis* in the supernatants of BA0711-treated cells. Lane 1 to 3, three different repeated samples in the CK group. Lane 4 to 6, three different repeated samples in the EBA12 group. Lane M, D2000 maker.

*In vitro*-cultured SLCs exhibited a typical fibroblast-like morphology, with most cells appearing as fusiform or irregular polygons (Figure 1A). The cell nuclei were round or oval and were often located centrally or slightly off-center. Besides, the cell boundaries were well-defined, uniform, and consistent. To verify the species of the strain, PCR was conducted using the primers that amplify the *16S rRNA* of *B. melitensis*. The product length was between 500 bp and 750 bp (Figure 1B), which was consistent with the expected amplification length of 732 bp for the primer. Furthermore, all supernatants from the experimental group (EBA12) tested positive for the above PCR assay (Figure 1C). In comparison, these bands were not appeared in the corresponding samples of the control group (CK). The BA0711-treated SLCs was prepared and verified, which could be used for the further transcriptome sequencing and analysis.

### 3.2 Sequencing and differential expression analysis

**Figure 2:**
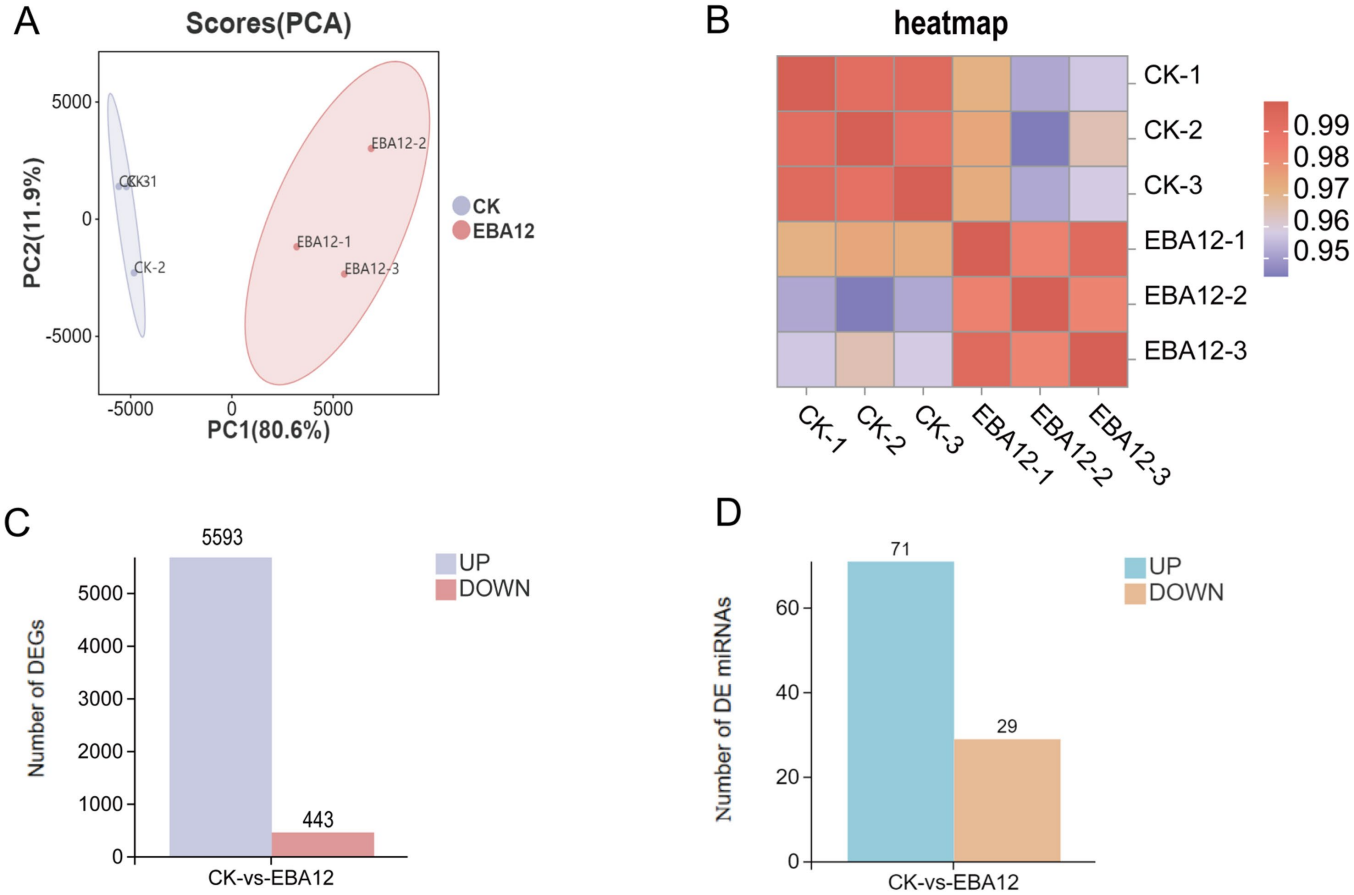
Sample correlation analysis and inter-group DEGs statistics **(A)** Principal component analysis (PCA) plot of sequencing samples. The samples of CK and EBA12 are represented by blue and red dots, respectively. **(B)** Correlation heatmap of sequencing samples. **(C)** Bar graph of DEGs in CK *vs* EBA12. **(D)** Bar graph of DE miRNAs in CK *vs* EBA12.

The obtained sequencing data of the RNA samples from the CK group and the EBA12 group were list in Table 1. The proportion of clean reads aligned to GCF_016772045.2 was over 97%. GC bases ratios were approximately 50%. Q30 base ratios, which represent base calling error rate of 0.1%, exceeded 94% in each sample. PCA plot manifested that treatment with BA0711 elicited a clear deviation of the EBA12 group samples from those of the CK group (Figure 2A). The first and second principal component accounted for 80.6 and 11.9% of the total variance, respectively. Moreover, the correlation heatmap revealed a strong correlation among samples within each group (Figure 2B). According to the screening criteria set above, 6,036 DEGs were identified in EBA12 compared to CK (CK vs EBA12), including 5,593 up-regulated DEGs and 443 down-regulated DEG (Figure 2C). A total of 100 DE miRNAs were screened in CK vs EBA12, most of which were also up-regulated (Figure 2D).

**Table 1:** Statistics of the main sequencing data of the samples.

Sample name	Raw reads	Clean reads	Mapped reads	GC bases ratio	Q30 bases ratio
CK-1	41,726,508	41,687,522	40,690,022 (97.61%)	49.46%	96.53%
CK-2	37,498,778	37,462,864	36,509,854 (97.46%)	49.97%	95.18%
CK-3	35,991,538	35,956,396	35,013,811 (97.38%)	48.47%	95.17%
EBA12-1	48,085,958	48,042,950	46,757,898 (97.33%)	49.30%	94.81%
EBA12-2	43,740,184	43,699,076	42,518,571 (97.30%)	49.29%	95.24%
EBA12-3	40,473,824	40,439,996	39,400,046 (97.43%)	50.04%	95.06%

The ratio of mapped reads (excluding rRNA-mapped reads), GC bases, and Q30 bases all refer to their proportions within the clean reads.

### 3.3 GO and KEGG enrichment analyses

**Figure 3:**
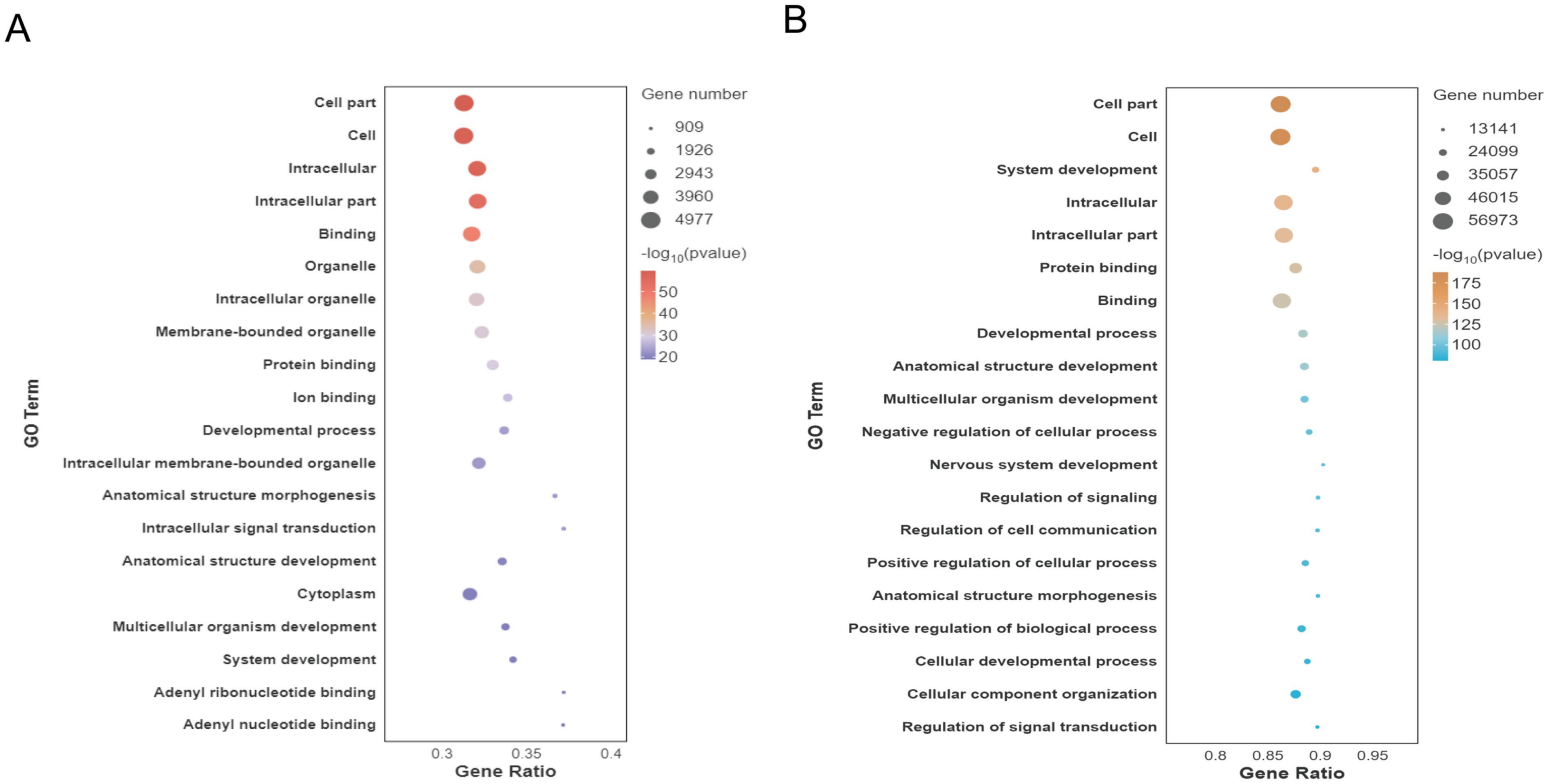
GO enrichment analysis of DEGs and the target genes of DE miRNAs. **(A)** GO enrichment scatter plot of DEGs in CK *vs* EBA12. **(B)** GO enrichment scatter plot of the target genes of DE miRNAs in CK *vs* EBA12.

**Figure 4:**
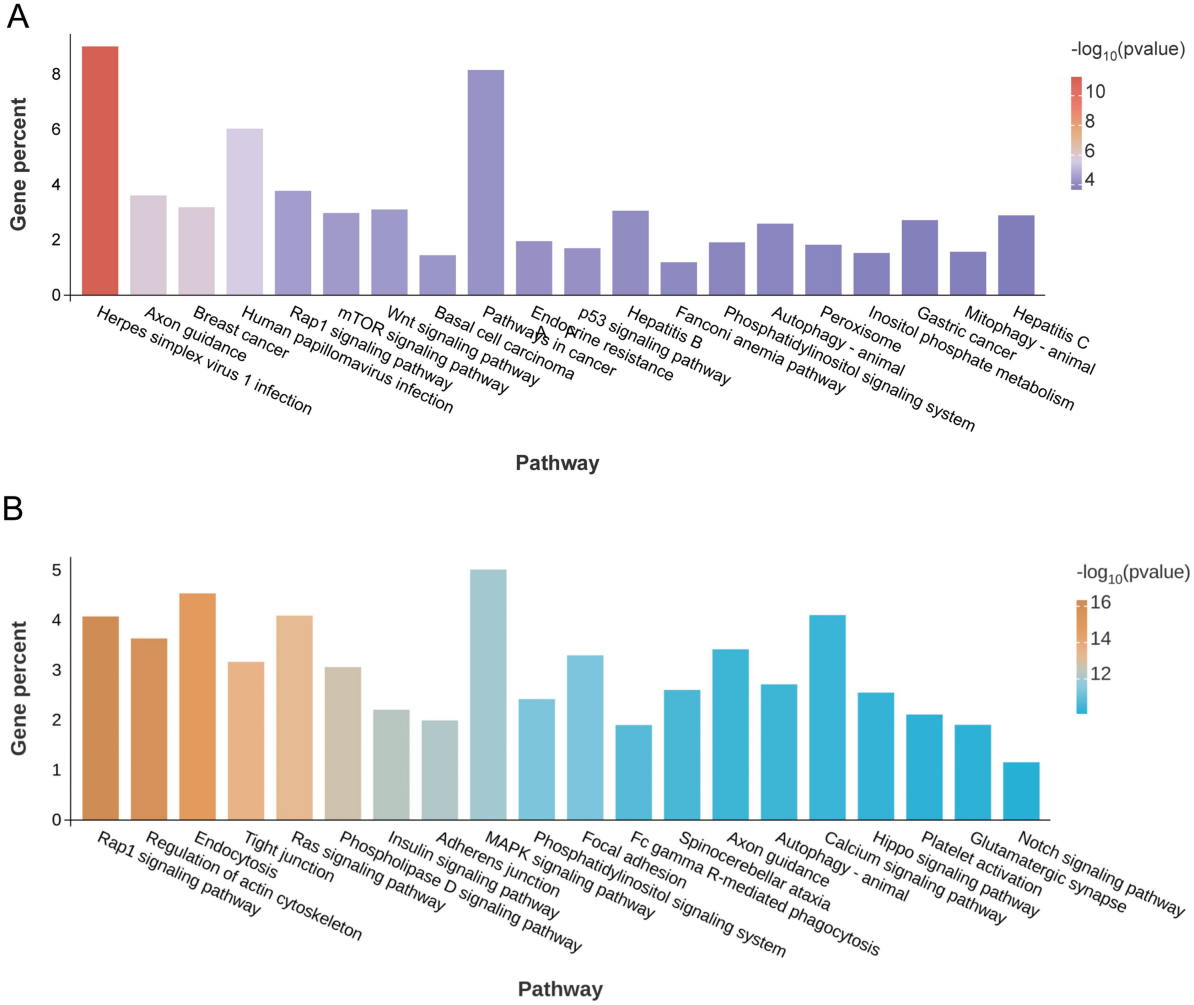
KEGG enrichment analysis of DEGs and the target genes of DE miRNAs. **(A)** KEGG enrichment bar plot of DEGs in CK vs. EBA12. **(B)** KEGG enrichment bar plot of the target genes of DE miRNAs in CK vs. EBA12.

GO enrichment analysis showed that DEGs were mainly enriched in cell part, cell, intracellular, intracellular part, binding, and protein binding (Figure 3A). The above mentioned GO terms were also related to the target genes of DE miRNAs (Figure 3B). The gene ratio of herpes simplex virus 1 infection was relatively higher than other DEGs-enriched pathways (Figure 4A). It was speculated that the BA0711-induced response in SLCs was possibly similar to that triggered by herpes simplex virus 1. Immune-related pathways were significantly by the DEGs, including Rap1 signaling pathway, mTOR signaling pathway, and autophagy-animal (Figure 4A and Supplementary Table S7). Moreover, the autophagy-animal pathway was also found in the top 20 significant pathways enriched by the target genes of DE miRNAs (Figure 4B).

### 3.4 Identification of hub DEGs in the autophagic pathway

**Figure 5:**
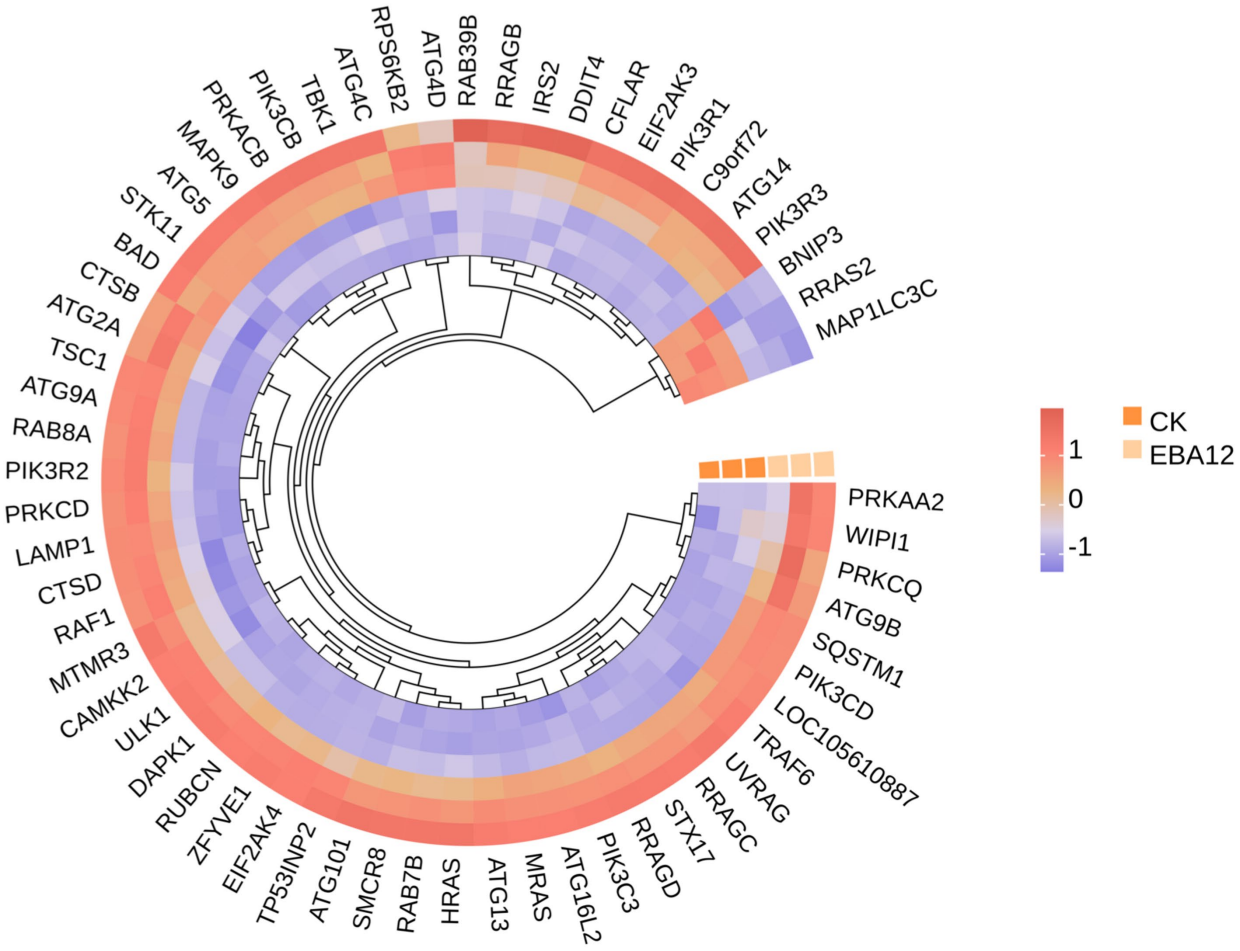
Heatmap of DEGs in the autophagy-animal pathway. The colored bar from red to blue indicates normalized expression levels of DEGs from up-regulation to down-regulation. The same colored blocks represent three different repeated samples in the CK or the EBA12 group.

Autophagic pathway was significantly enriched by both DEGs and the target genes of DE miRNAs. Besides, previous studies have found that autophagy contributes to the immunopathogenesis of *Brucella* (35). Therefore, the expression of the DEGs in the autophagy-animal pathway were investigated. The results showed that most of DEGs in this pathway were up-regulated after BA0711 stimulation (Figure 5). Among them, ULK1, ATG13, ATG14, PIK3C3, ATG5, ATG101, and ATG16L2 were identified as the top 7 proteins by the EPC algorithm within the cytoHubba plugin (Supplementary Figure S1; Supplementary Table S1).

### 3.5 miRNA-mRNA regulatory network

**Figure 6:**
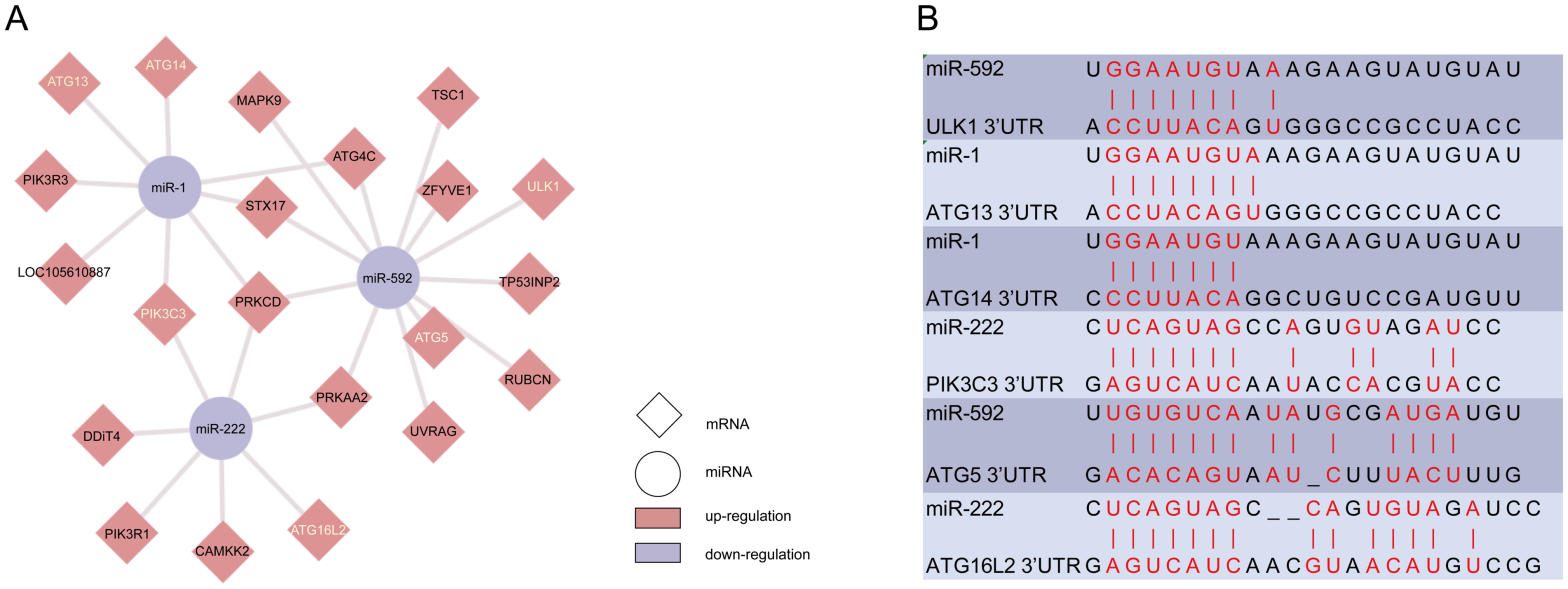
Analysis of miRNA-mediated regulation of autophagic genes. **(A)** DE miRNA-DEGs regulatory relationship of the autophagy-animal pathway. The mRNAs highlighted in white encode the hub proteins in Supplementary Figure S1. **(B)** Prediction of miRNA bingding sites on the 3′-UTR of *ULK1*, *ATG13*, *ATG14*, *PIK3C3*, and *ATG5* mRNA using miRanda and TargetScan.

The target genes of DE miRNAs involved in the autophagic pathway. It was speculated that miRNAs act as regulatory factors of BA0711-induced autophagy in SLCs. Accordingly, the DE miRNA-DEGs regulatory relationship related to the autophagy-animal pathway was constructed. Six DEGs (ULK1, ATG13, ATG14, PIK3C3, ATG5, and ATG16L2) encoding hub proteins in the PPI network were found to be potentially regulated by miRNAs (Figure 6A). Among them, ULK1 and ATG5 were predicted to be regulated by miR-592. ATG13 and ATG14 may be associated with miR-1. Moreover, miR-222 possibly regulated ATG16L2 and PIK3C3 (Figure 6B).

### 3.6 RT-qPCR validation of autophagic DEGs and DE miRNA

**Figure 7:**
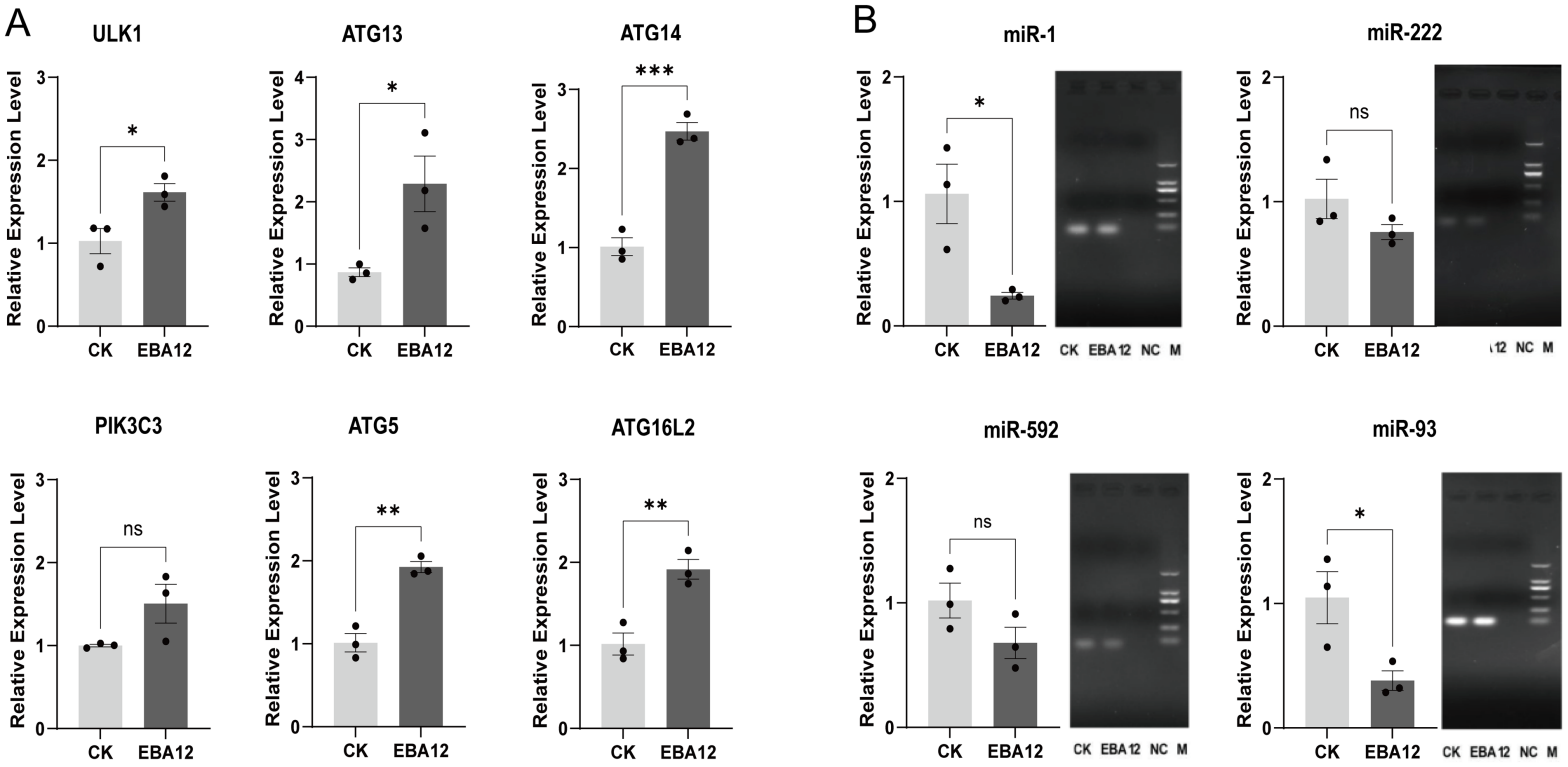
RT-qPCR validation of autophagy-related DEGs and DE miRNAs. **(A)** RT-qPCR validation of hub DEGs in the autophagy-animal pathway. EBA12 and CK represent RNA samples from EBA12 and CK, respectively. **(B)** RT-qPCR validation of miR-1, miR-222, miR-592, and miR-93. Left: Relative expression of miRNAs after BA0711 stimulation. Right: Agarose gel electrophoresis results of the corresponding RT-qPCR products. Lane CK: CK. Lane EBA12: EBA12. Lane NC: negative control. Lane M: D2000 Marker. The results were represented as mean ± standard error.

Among the hub DEGs in the autophagy-animal pathway, ULK1, ATG13, ATG14, PIK3C3, ATG5, and ATG16L2 were predicted to be regulated by DE miRNA. Therefore, the expression of these DEGs and corresponding DE miRNAs (including miR-592, miR-1, and miR-222) were verified by RT-qPCR. The results showed that ULK1, ATG13 (*p* < 0.05) and ATG14 (*p* < 0.001), ATG5, ATG16L2 (*p* < 0.01) were significantly up-regulated in the BA0711-treated SLCs (Figure 7A). MiR-1 was significantly down-regulated in CK vs EBA12 (*p* < 0.05, Figure 7B). In addition, miR-93, a potential marker of chronic *Brucella* infection (36, 37), was also significantly down-regulated in CK vs EBA12, which is consistent with previous research.

### 3.7 Regulation validation of miRNAs

**Figure 8:**
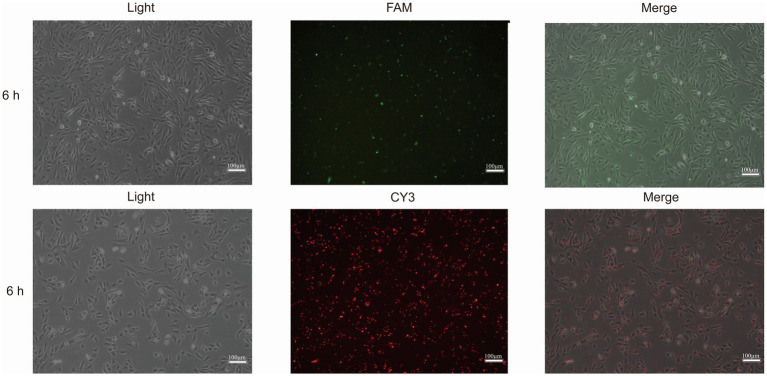
Transfection of scrambled miRNA mimics and inhibitor (Scale bar = 100 μm). Upper row and lower row represent SLCs transfected with FAM and CY3 labeled miRNA inhibitor and mimics, respectively.

**Figure 9:**
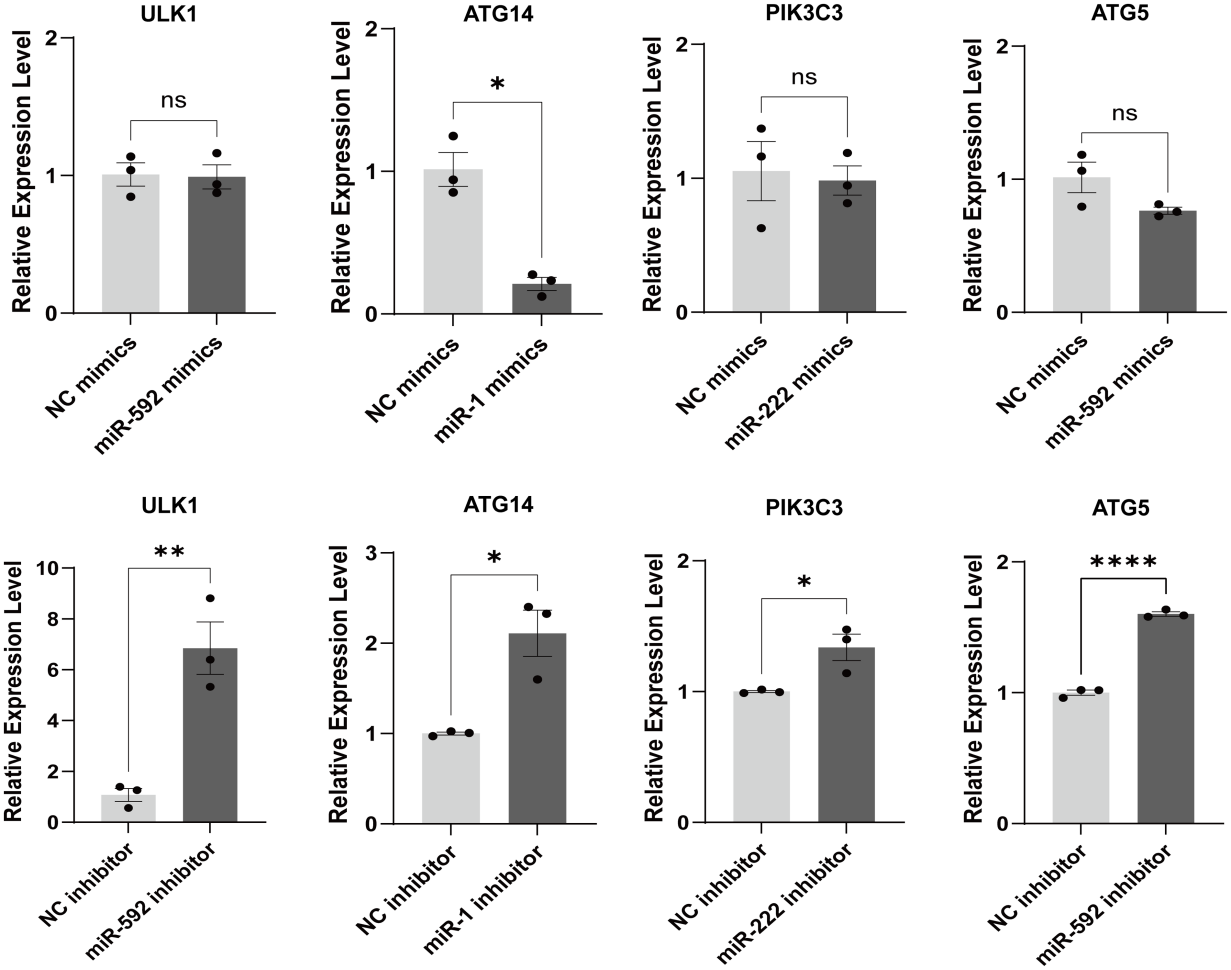
Regulatory valadaition of miRNA mimics and inhibitors. Gray bars represent the negative control (NC mimics or NC inhibitors) group, while black bars indicate the miRNA treatment group (mimics or inhibitors). All data are presented as the mean ± standard error. Three independent biological replicates were performed for each group, and each sample was run in triplicate (three technical replicates) in the RT-qPCR assays. The Y-axis represents the relative expression level normalized to the reference gene. Statistical significance was determined using an unpaired *t*-test. ns, no significant difference. *p < 0.05, **p < 0.01, ****p < 0.0001.

The DE miRNA-DEGs regulatory relationship indicated potential cross-talk between miRNAs and autophagy-related genes. Therefore, we transfected mimics and inhibitors of these miRNAs to verify whether the corresponding hub genes are regulated. Fluorescence signals from the scrambled miRNA inhibitor and mimics reflected the transfection efficiency of the mimics and inhibitors targeting the hub genes (Figure 8). The RT-qPCR data revealed that miR-1 negatively regulated ATG14 mRNA levels, as evidenced by significant downregulation in miR-1 overexpression experiments (*p* < 0.05) and corresponding upregulation in miR-1 knockdown assays (*p* < 0.05) (Figure 9).

### 3.8 Results of dual-luciferase reporter assay

**Figure 10:**
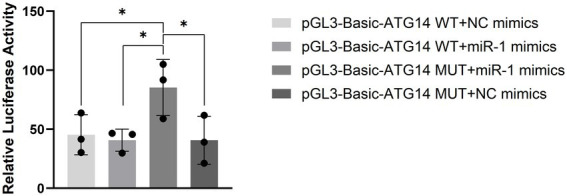
Results of dual-luciferase reporter assay. *p < 0.05. The results were represented as mean ± standard error.

As shown in Figure 6B, consecutive binding sites between miR-1 and the 3’UTR of ATG14 imply that miR-1 targets ATG14. Dual-luciferase reporter assay results (Figure 10) revealed that luciferase activity of WT pGL3-Basic-ATG14 was significantly decreased compared with the MUT group upon miR-1 mimics transfection (p < 0.05), confirming that miR-1 binds to the ATG14 3’UTR and exerts inhibitory effects. No obvious difference was detected between WT and MUT under NC mimics treatment, excluding non-specific interference caused by vector mutation. These findings verify that ATG14 is a direct target of miR-1.

## 4 Discussion

Eukaryotes utilize autophagy to degrade intracellular substances, thus maintaining homeostasis and modulating metabolism and immunity (36). Although autophagy helps eliminate intracellular bacteria, *Brucella* exploit this process to form autophagic *Brucella*-containing vacuoles (BCVs) to promote infection. Compared with the parental strain, *B. abortus* A19Δ*virB* induced a higher level of autophagy in mouse macrophages and dendritic cells (38). Prior studies have demonstrated that the *B. melitensis* M5-90 mediates cellular autophagy by targetedly regulating the *Tbc1d14* gene via miR-146b-5p in RAW264.7 macrophages (17). Accordingly, in the present study, we selected the live *B. melitensis* BA0711 as the research subject to investigate the regulatory network mechanism underlying autophagy induced in SLCs.

Analysis of the DEGs revealed significant enrichment of pathways related to autophagy, including the autophagy pathway, as well as the lysosome and mTOR signaling pathways (Supplementary Table S7). Within the autophagy pathway, the majority of DEGs were upregulated, and all genes in the lysosome pathway also showed increased expression (Figure 6 and Supplementary Figure S2). Notably, while genes involved in autophagosome formation and elongation, such as *ULK1*, *ATG13*, *ATG14*, *ATG5*, and *ATG16L2*, were significantly upregulated in the autophagy pathway, the *MTOR* gene itself did not show significant alteration (Supplementary Table S4). This observation suggests that BA0711-induced autophagy in SLCs might operate through an mTOR-independent mechanism. Further investigation into upstream regulators or alternative signaling inputs would be required to confirm this.

Subsequent prediction analysis utilizing the EPC algorithm of the cyto Hubba plugin identified *ULK1*, *ATG13*, *ATG14*, *PIK3C3*, *ATG5*, *ATG101*, and *ATG16L2* as the top 7 key proteins within the autophagy pathway. Consistent with their predicted roles in autophagosome biogenesis, RT-qPCR confirmed the significant upregulation of *ULK1*, *ATG5*, *ATG13*, *ATG14*, and *ATG16L2* in EBA12-infected SLCs compared to the CK. These proteins are integral to the formation of autophagosomes: ULK1, in complex with ATG13, ATG14, and ATG101, initiates phagophore formation (39, 40); In addition, ATG14 participates in the assembly of the Class III PI3K Complex I and plays a critical regulatory role in the early stage of autophagosome formation (41). ATG5, as a core component of the ATG12-5-16 L1 complex, is crucial for phagophore elongation (42); and ATG16L2 has been shown to positively regulate autophagy by promoting the assembly of this complex (43). Furthermore, the potential clinical relevance of these findings is underscored by a genome-wide association study in goats that identified a single nucleotide polymorphism within the ATG16L2-containing genomic region associated with brucellosis occurrence (44). Therefore, the upregulated autophagic DEGs identified here, particularly *ULK1*, *ATG5*, *ATG13*, *ATG14*, and *ATG16L2*, are likely crucial players in BA0711-induced autophagy in SLCs. In addition to autophagy-related pathways, the DEGs also significantly enriched other immune-relevant pathways, including the MAPK, TNF, and NOD-like receptor signaling pathways (Supplementary Table S7). These pathways are known to be involved in innate immune responses and host defense. Their co-enrichment with autophagy pathways suggests a complex interplay where BA0711 may activate multiple defense arms within SLCs simultaneously, potentially through synergistic signaling cascades that also involve or are influenced by autophagic processes. Future studies could explore how these pathways interact with and potentially modulate BA0711-induced autophagy in SLCs.

Through integrated analysis of mRNA-miRNA sequencing data coupled with RT-qPCR validation, we identified three autophagy-related DE miRNAs (miR-1, miR-222, and miR-592) in BA0711-infected SLCs, establishing a potential regulatory network. MiR-1, while predominantly known for muscle development (45, 46), has been implicated in bacterial infections through its potential to modulate antigen presentation (40) and serve as a diagnostic marker (47). MiR-222, known for its pleiotropic effects, has been linked to inflammatory responses and innate immune memory, with reported roles in regulating inflammatory cytokine production and interferon induction (48–50). MiR-592 has been associated with cell signaling pathways and tumorigenesis (51, 52), and its involvement in viral infections has also been noted (53). Upon transfection with the corresponding miRNA mimics, only miR-1 significantly downregulated ATG14, whereas mimics of miR-592 and miR-222 failed to induce significant changes in their targets. This asymmetric phenomenon—where inhibition is effective but overexpression is not—suggests a complex regulatory environment within SLCs. A plausible explanation is the existence of high-affinity complementary sequences (such as miRNA sponges or circRNAs) that act as competing endogenous RNAs (ceRNAs) to sequester the exogenously introduced mimics, thereby interfering with their intended regulatory functions (45).

The current investigation of the above three DE miRNA target genes (ULK1, ATG14, PIK3C3, and ATG5) reveals the complexity of these pathways and the cell-type-specific nature of miRNA action. *ATG14* is inhibited by miR-15b-5p and miR-424-5p in endothelial cells (54, 55), *PIK3C3* is regulated by miR-4270 (56) and miR-34a-3p (57), and *ULK1* is regulated by miR-106a and miR-665 in *mycobacterial* infections (58, 59). Through systematic screening, we identified functionally relevant miRNA-mRNA interaction networks during BA0711 infection, with experimental validation confirming miR-1-mediated suppression of ATG14 expression – a key autophagy regulator. The prior report on *B. melitensis* M5-90 regulating *Tbc1d14* via miR-146b-5p in macrophages further supports the notion that distinct *B. melitensis* and host cell types may employ different miRNA-mediated autophagy regulatory mechanisms (17).

In conclusion, this study has identified a novel miRNA-mRNA regulatory network governing *B. melitensis* BA0711-induced autophagy in SLCs. We found that key autophagic genes, including *ULK1*, *ATG5*, *ATG13*, *ATG14* and *ATG16L2*, are upregulated, suggesting an mTOR-independent autophagic response. Furthermore, we unveiled a specific miRNA-mRNA axes (miR-1-*ATG14*) that likely play a regulatory role in this process. The observed cell-type specific regulatory characteristics of miRNA-mediated autophagy highlight the intricate mechanisms by which host cells interact with *Brucella* infection. These findings offer a novel perspective on the molecular interactions between live *Brucella* vaccines and host cells and lay the groundwork for further experimental validation and potential therapeutic development. It is important to note a technical limitation in the characterization of autophagy in this study. Although our transcriptomic data suggest the activation of autophagic pathways, protein-level validation via Western blotting was challenging due to the lack of commercially available antibodies specific to sheep. Future experiments may involve attempting validation using antibodies raised in other species.

## 5 Conclusion

In this study, transcriptome sequencing revealed the mRNA and miRNA profiles of BA0711-treated host reproductive cells (SLCs). Five hub autophagy-related DEGs, including *ULK1*, *ATG13*, *ATG14*, *ATG5*, and *ATG16L2*, were validated by RT-qPCR. Overexpression and knockdown experiments of miR-1, together with dual-luciferase reporter assays, demonstrated that miR-1 inhibits the mRNA expression of its downstream target ATG14 by binding to the 3’UTR of ATG14. This suggests that the miRNA-mediated autophagic regulatory pathway induced by live *Brucella* vaccine strains may exhibit cell type-specific differences between immune cells and germ cells.

## Data Availability

The datasets presented in this study can be found in online repositories. The data presented in this study have been stored in the Genome Sequence Archive repository (https://ngdc.cncb.ac.cn/gsa, accession number: CRA034698).

## Footnotes

1
http://www.r-project.org/

## Supplementary material

SUPPLEMENTARY FIGURE S1 Hub proteins in the autophagy-animal pathway predicted by EPC algorithm.

SUPPLEMENTARY FIGURE S2 DEGs expression plot of the lysosome pathway.

SUPPLEMENTARY TABLE S1 Differentially expressed genes (DEGs) in the autophagy pathway.

SUPPLEMENTARY TABLE S2 PPIs of DEGs in the autophagy pathway.

SUPPLEMENTARY TABLE S3 RT-qPCR primers of DEGs.

SUPPLEMENTARY TABLE S4 RT-qPCR primers of miRNAs.

SUPPLEMENTARY TABLE S5 Reverse transcription primers of miRNAs.

SUPPLEMENTARY TABLE S6 Sequence of miRNA mimics and inhibitor.

SUPPLEMENTARY TABLE S7 Immune-related pathways enriched by the DEGs in CK *vs* EBA12.
