# Supplementary figures and images for: MiR-1 mediates autophagy via ATG14 in sheep Leydig cells infected with Brucella melitensis strain BA0711
Source: Front Vet Sci. 2026 Jun 16;13:1809720. doi: 10.3389/fvets.2026.1809720 (PMC13314496; doi:10.3389/fvets.2026.1809720)

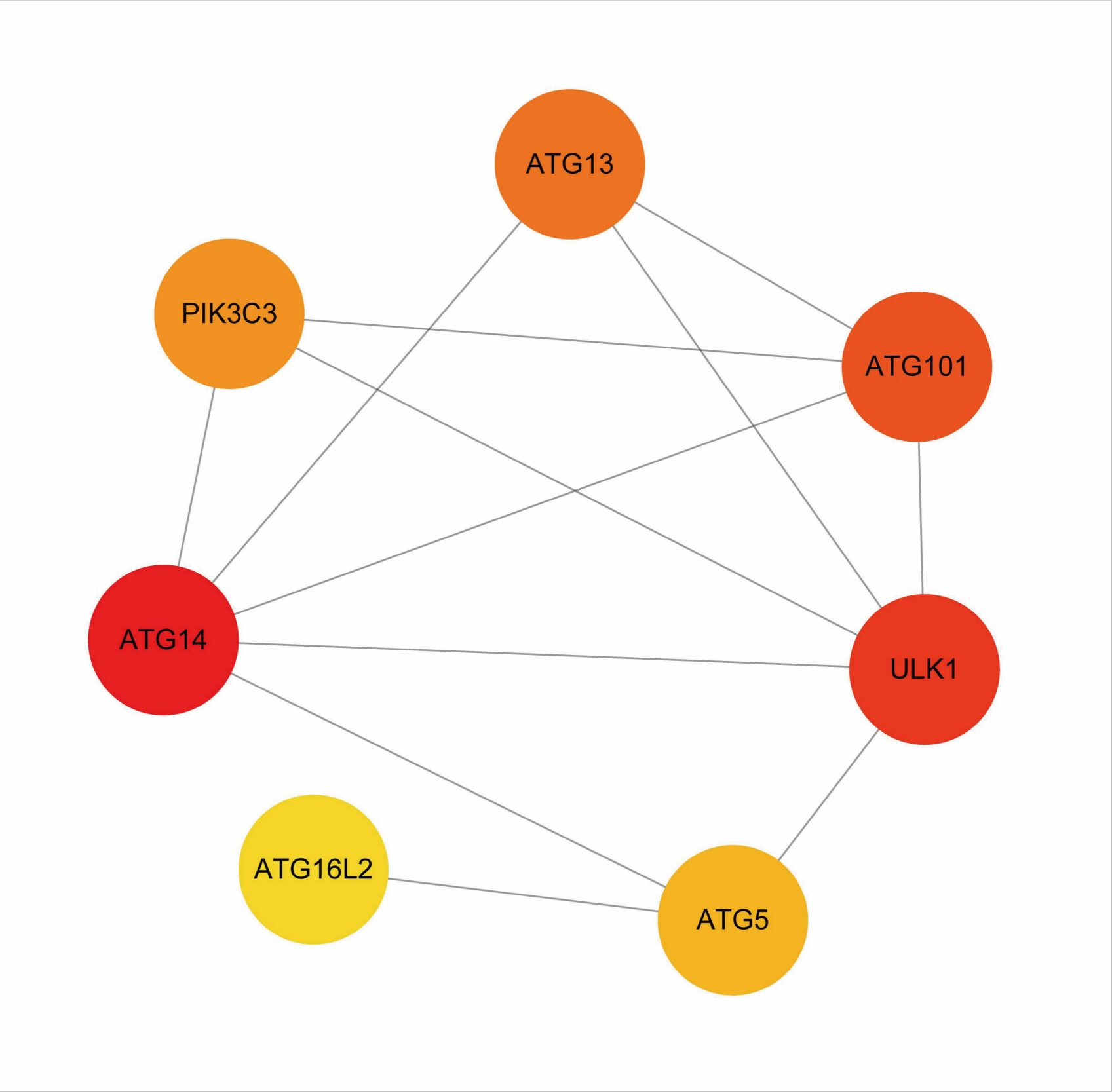

Supplement: SUPPLEMENTARY FIGURE S1 — Hub proteins in the autophagy-animal pathway predicted by EPC algorithm. [file Image_1.TIFF]

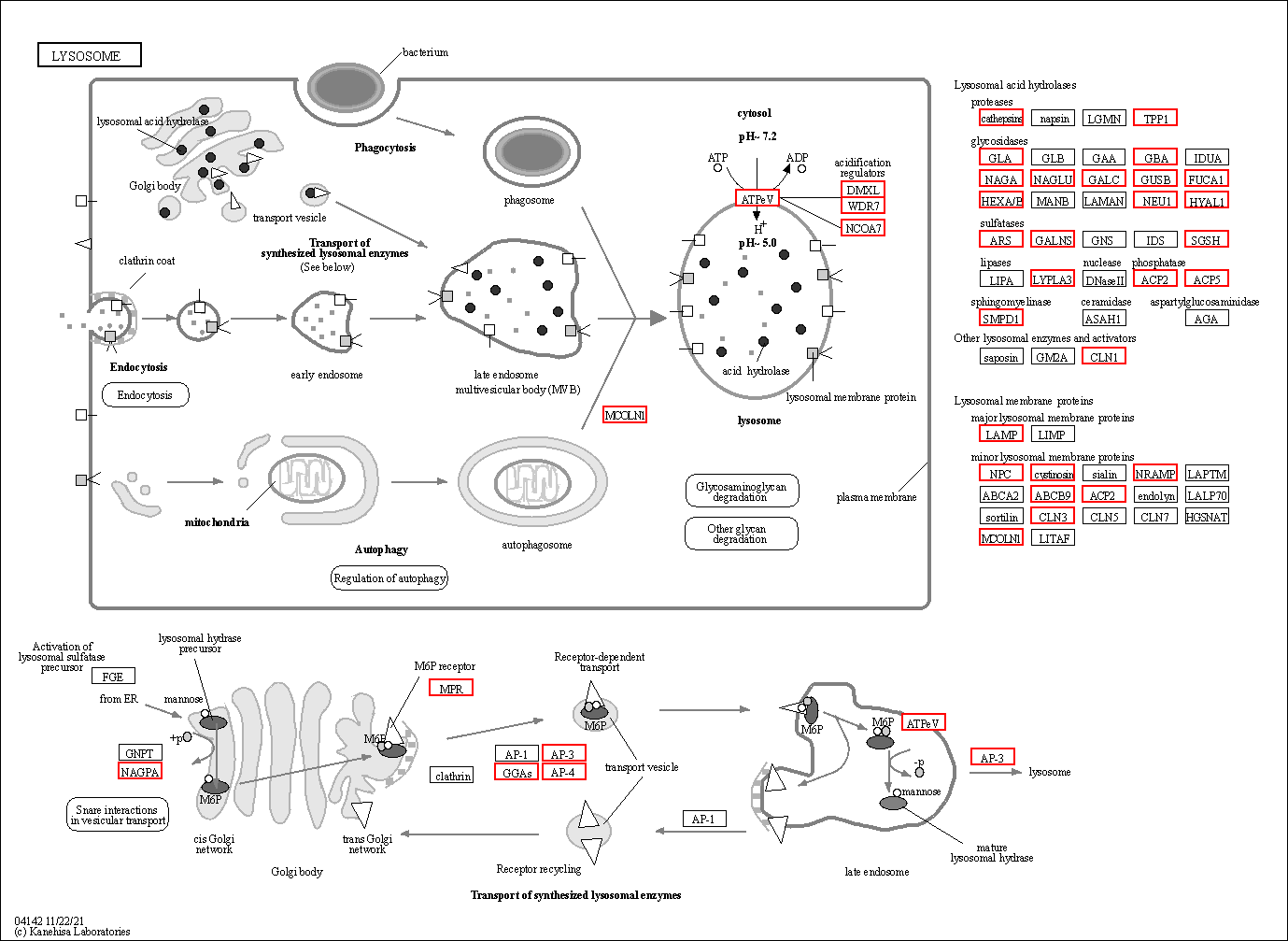

Supplement: SUPPLEMENTARY FIGURE S2 — DEGs expression plot of the lysosome pathway. [file Image_2.PNG]
